# Gentulizumab, a novel anti-CD47 antibody with potent antitumor activity and demonstrates a favorable safety profile

**DOI:** 10.1186/s12967-023-04710-6

**Published:** 2024-03-01

**Authors:** Tao Wang, Si-Qin Wang, Yin-Xiao Du, Dan-Dan Sun, Chang Liu, Shuang Liu, Ying-Ying Sun, Hai-Long Wang, Chun-Sheng Zhang, Hai-Long Liu, Lei Jin, Xiao-Ping Chen

**Affiliations:** 1 Department of Clinical Pharmacology, Xiangya Hospital, Central South University, Changsha, 410008 Hunan People’s Republic of China; 2 GeneScience Pharmaceuticals Co., Ltd, Changchun, 130012 Jilin People’s Republic of China; 3 National Clinical Research Center for Geriatric Disorders, Xiangya Hospital, Central South University, Changsha, 410008 Hunan People’s Republic of China

**Keywords:** Cancer immunotherapy, CD47, Pyroglutamic acid, Phagocytosis

## Abstract

**Background:**

Targeting CD47/SIRPα axis has emerged as a promising strategy in cancer immunotherapy. Despite the encouraging clinical efficacy observed in hematologic malignancies through CD47-SIRPα blockade, there are safety concerns related to the binding of anti-CD47 antibodies to CD47 on the membrane of peripheral blood cells.

**Methods:**

In order to enhance the selectivity and therapeutic efficacy of the antibody, we developed a humanized anti-CD47 monoclonal antibody called Gentulizumab (GenSci059). The binding capacity of GenSci059 to CD47 was evaluated using flow cytometry and surface plasmon resonance (SPR) methods, the inhibitory effect of GenSci059 on the CD47-SIRPα interaction was evaluated through competitive ELISA assays. The anti-tumor activity of GenSci059 was assessed using in vitro macrophage models and in vivo patient-derived xenograft (PDX) models. To evaluate the safety profile of GenSci059, binding assays were conducted using blood cells. Additionally, we investigated the underlying mechanisms contributing to the weaker binding of GenSci059 to erythrocytes. Finally, toxicity studies were performed in non-human primates to assess the potential risks associated with GenSci059.

**Results:**

GenSci059 displayed strong binding to CD47 in both human and monkey, and effectively inhibited the CD47-SIRPα interaction. With doses ranging from 5 to 20 mg/kg, GenSci059 demonstrated potent inhibition of the growth of subcutaneous tumor with the inhibition rates ranged from 30.3% to complete regression. Combination of GenSci059 with 2.5 mg/kg Rituximab at a dose of 2.5 mg/kg showed enhanced tumor inhibition compared to monotherapy, exhibiting synergistic effects. GenSci059 exhibited minimal binding to hRBCs compared to Hu5F9-G4. The binding of GenSci059 to CD47 depended on the cyclization of N-terminal pyroglutamic acid and the spatial conformation of CD47, but was not affected by its glycosylation modifications. A maximum tolerated dose (MTD) of 450 mg/kg was observed for GenSci059, and no significant adverse effects were observed in repeated dosages up to 10 + 300 mg/kg, indicating a favorable safety profile.

**Conclusion:**

GenSci059 selectively binds to CD47, effectively blocks the CD47/SIRPα axis signaling pathway and enhances the phagocytosis effects of macrophages toward tumor cells. This monoclonal antibody demonstrates potent antitumor activity and exhibits a favorable safety profile, positioning it as a promising and effective therapeutic option for cancer.

**Supplementary Information:**

The online version contains supplementary material available at 10.1186/s12967-023-04710-6.

## Background

Despite the development of various anticancer drugs based on different strategies, the high morbidity and mortality of tumors remain a significant challenge [1–3]. Macrophage is an essential component of the innate immune system and has been shown to play a crucial role in anti-tumor responses. One promising treatment in cancer therapy is targeting CD47, a transmembrane glycoprotein widely expressed on the surface of various cells. Signal regulatory protein α (SIRPα) is the endogenous receptor of CD47 expressed in macrophage [4, 5]. The CD47-SIRPα complex mediates bidirectional signal transduction that is essential for various biological processes such as immune response, macrophage phagocytosis, hematopoietic stem cell suppression, and central nervous system development [6–11].

CD47 is expressed ubiquitously on the membrane of various types of cancer, including breast cancer, leiomyosarcoma, myeloma, osteosarcoma, non-Hodgkin’s lymphoma, leukemia, and hepatocellular carcinoma [12–19]. CD47 expressed on the surface of tumor cells binds to SIRPα on the surface of macrophages, leading to phosphorylation of the immune receptor tyrosine inhibitory motif (ITIM) in the intracellular domain of SIRPα. Consequently, Src homology phosphatase (SHP) is dephosphorylated and the downstream signaling pathways such as Syk and PI3K were inhibited, which leads to the inhibition of macrophage phagocytosis to tumor cells [20–22]. This CD47-SIRPα interaction, known as the “don’t eat me” signal, allows cancer cells to escape immune surveillance [12, 23–25]. Anti-CD47 antibodies have been the focus of drug development in current immunotherapy efforts, as they inhibit the CD47-SIRPα interaction and promote cancer cell destruction by macrophages. This phagocytosis can stimulate the presentation of tumor-specific antigens, recruitment of specific killer T cells, and inhibition of tumor growth and metastasis [4, 26]. Several CD47-targeting drugs are currently under clinical investigation, including Hu5F9-G4 [27, 28], CC-90002 [29], and TTI-621 [30]. However, safety issues associated with these agents have emerged. For instance, Hu5F9-G4 and TTI-621 can cause acute anemia and thrombocytopenia in humans [27, 28, 30]. The cyclization of CD47 N-terminal pyroglutamate on the tumor cell surface can enhance its binding ability to SIRPα [31, 32]. Given that anti-CD47 antibodies and SIRPα-Fc fusion proteins contribute to these side effects, the toxicity of anti-CD47 antibodies is assumed to be Fc-dependent. Therefore, a major controversy in the development of current anti-CD47 therapies is whether the therapeutic effect is due to the specific of CD47 inhibition or non-specific antibody-dependent cell-mediated cytotoxicity (ADCC).

In this study, we designed a novel anti-CD47 antibody, Gensci059, which distinguishes itself from other CD47 targeting agents by lacking hematological liabilities while retaining its anti-tumor properties.

## Materials and methods

### Monoclonal antibody generation and humanization

Generation of monoclonal antibodies targeting human CD47 followed a systematic procedure. Initially, a gene fragment comprising the extracellular region of human CD47 and hIgG1Fc was constructed. The DNA fragment was subsequently cloned into the pcDNA3.1 vector using the Hind III/EcoR I restriction enzyme cutting site, resulting in the creation of an expression plasmid. The expression plasmid was transiently transfected into 293F cells, and the expressed proteins were purified employing a protein purification liquid chromatography system (AKTA purifier 10, GE Healthcare) in accordance with the manufacturer’s provided protocol. Immunization of Balb C mice involved the use of CD47-hFC fusion protein alongside a specific adjuvant formulation and immunization regimen. Splenocytes obtained from immunized mice were collected and subsequently utilized in a hybridoma experiment in conjunction with P3X63Ag8.653 myeloma cells. The splenocytes and myeloma cells were co-cultured with a ratio of 5:1 in HAT medium to generate hybrid cell lines. Two weeks after the fusion, cell supernatants were collected for ELISA and functional assays. Positive hybridoma cell lines were selected and cultured for 7 days, the supernatants were collected and subsequently subjected to purification via protein A affinity chromatography. From the monoclonal cell lines, the monoclonal antibody with the highest potential (designated as clone number 1.43.1) was selected through a comprehensive screening process. To humanize the mouse-derived anti-human CD47 mAb (1.43.1), complementarity-determining region (CDR) residues were grafted onto a human germline framework. Five sets of suitable framework region (FR) sequences were identified, with possible post-translational modification (PTM) motifs removed. A total of 25 combinations were generated by combining five heavy chain vectors and five light chain vectors, followed by transient expression in 293E cells. The expression quantity and binding properties of these 25 combinations were evaluated. The final candidate antibody was transiently expressed using 293E cells in Freestyle medium. The supernatant was filtered through a 0.22 μm filter membrane, followed by passage through the MabSelect SuRe affinity chromatography column (GE Healthcare). Selected humanized antibodies were obtained and named Gensci059. The purified antibodies were then stored at 4 °C for subsequent use. The Hu5F9-G4 antibody employed in this study as a control was also synthesized in our laboratory, referencing the sequence described in published literature. The synthesized antibody matched the sequence detailed in as described [33].

### Affinity measurement

For cell binding analysis, CT26 cells were transfected and stably overexpressed human CD47 (hCD47) or cynomolgus monkey CD47 (cCD47). The overexpressed cells were incubated with indicated mAbs, bound antibodies were detected using Goat Anti-Human IgG Fc FITC (Jackson) by flow cytometry analysis (FACS). The KD value was determined using the following formula: Y = Bmax*X/(Kd + X) [Y: bound IgG, X: Free IgG]. The affinity of GenSci059 to CD47 was assessed using surface plasmon resonance (SPR) on a Biacore T200 system, employing a recombinant human, cynomolgus monkey, mouse and rat CD47ECD/His fusion protein. The processes of molecular binding (120 s) and dissociation (300 s) were recorded using Biacore control software V.2.0. The data were analyzed with a 1:1 model using Biacore T200 Evaluation V.2.0.

### Blocking of SIRPα to CD47

The ability of Gensci059 to disrupt recombinant SIRPα binding to CD47 was detected using a competitive ELISA assay. 1 µg/mL of SIRPα/His was premixed with various concentrations of Gensci059 (ranging from 0.003 to 20 µg/mL with threefold serial dilution). The mixture was then added into the well coated 0.5 µg/mL of CD47ECD/Fc fusion protein. The bound SIRPα/His protein was detected using anti-His-HRP conjugate (Proteintech), which was visualized with TMB Stabilized Chromogen (Sigma) and detected using an Multiskan™ FC (Thermo Fisher).

### Phagocytosis assay

The influence of CD47 antibody on phagocytosis of cells was investigated by fluorescence imaging or FACS. Primary peritoneal macrophages isolated and purified from C57BL/6 mice were stained with PKH26 dye one day in advance (4 μM, 5 min) and seeded in 96-well plates with 20,000 cells/well. The next day, the macrophages were resuspended in serum-free medium after washing and seeded on macrophages with 8 × 10^4^ cells/well after incubated at 37℃ for 2 h. Target cells were stained with 2 μM 5,6- carboxyfluorescein diacetate, succinimidyl ester (CFSE) dye for 10 min. The various antibodies were added to the cell mixture started at 100 µg/mL with fivefold dilution and 10 concentration points. After 2 h of culture, the pellets were resuspended in 400 µL PBS and analyzed for phagocytosis using FACS Canto™. Data were analyzed using Flowjo. The ratio of PKH + CFSE + double positive cells to PKH + positive cells was recognized as a phagocytosis index.

### Migration assay

For migration assay, we employed 6.5 mm diameter transwells with 8 μm pores (Corning 3422) as described in previously [34, 35],. One hundred thousand mononuclear leukemic or cell line cells, in 100 μL of RPMI-1640 medium containing 0% FBS, were added to the upper chamber, while the lower chamber was filled with 500 μL of medium containing 10% FBS. Gensci059 (final concentration: 10 μg/mL) or unrelated Human IgG4 were added into the upper chamber. Subsequently, leukemic cells were allowed to migrate at 37 °C for 24 h. After this incubation period, the inserts were carefully removed, and the cell density was determined using trypan blue staining and cell counting.

### Binding experiment with human blood cells

In order to assess the binding activity of the antibody with human blood cells, in vitro experiments were conducted. A total of 20 healthy human red blood cells (hRBCs), peripheral blood mononuclear cells (PBMCs), and platelets (PLTs), consisting of 2 × 10^5^ cells (100 μL) each, were added to individual wells of a 96-well plate. The cells were subsequently washed twice with a solution of 1% BSA/1xPBS. The initial concentration of the antibody was 100 μg/mL, which was then subjected to a tenfold dilution. The binding of the antibody to the blood cells was measured using flow cytometry analysis.

### Antibody-dependent cell-mediated cytotoxicity (ADCC) and complement-dependent cytotoxicity (CDC) assays

The ADCC assay used the constructed NK92/CD16a stable cell line as effector cells and Rituxan (rituximab, targeting CD20) as a positive control antibody. The antibody concentration was started at 20 µg/mL with tenfold dilution and 10 concentration points. The killing effect on target cells was detected using a lactate dehydrogenase (LDH-Glo™ Cytotoxicity Assay) kit (Promega). The complement of CDC was obtained from normal human serum (NHS) with a final serum concentration of 20%. The starting concentration was 20 μg/mL at tenfold dilution and 8 concentration points. Target cell viability was detected using a CellTiterGlo® Luminescent Cell Viability Assay kit (Promega).

### Liquid chromatography tandem mass spectrometry

Peptides were separated using an Agilent UPLC 1290 Infinity II Liquid Chromatography system (Agilent, USA) and introduced into the mass spectrometer. The mobile phases consisted of a 0.05% trifluoroacetic acid–water solution in phase A and a 0.05% trifluoroacetic acid-acetonitrile solution in phase B. Separation was achieved over an 80-min gradient. The samples were separated and detected using the following chromatographic gradient: 0 ~ 2 min, 0% B; 2 ~ 72 min, 0–37% B; 72 ~ 72.5 min, 37–90% B; 72.5 ~ 74 min, 90% B; 72.5 ~ 74.5 min, 0% B; 74.5 ~ 80 min, 0% B. The flow rate of the chromatographic column was set at 0.25 ml/min, the column temperature at 40 ℃, and the injection volume was 20 μL. Mass spectrometry data were collected in positive ion mode using a high-resolution time-of-flight mass spectrometer, Triple TOF 6600 (AB SCIEX, USA). The primary mass scan range covered m/z 200 to 1800. The instrument parameters were as follows: the curtain gas flow rate was 35 arb; auxiliary gas flow rate 50 arb; nebulizer gas flow rate 50 arb; nebulizer gas temperature 550 ℃; accumulation time 0.5 s; ion spray voltage 5500 V; declustering potential 80 V. Raw data files from the LC–MS mass spectrometry analysis were collected using PeakView (version 2.2, AB SCIEX, USA) for subsequent data retrieval and analysis.

### Selected binding and epitope analysis

For Gensci059 binding epitope prediction, based on the sequence of Gensci059, the Fv of Gensci059 was constructed using MODELLER in Accelrys Discovery Studio 2017 (BIOVIA, San Diego, CA, USA) and protein docking using ZDOCK in Accelrys Discovery Studio 2017 (BIOVIA, San Diego, CA, USA). The CD47 crystal structure Hu5F9-G4 (PDB ID:5IWL) was used as a receptor protein. All the Gensci059-CD47 complex MD simulations, with CHARMM force field, were carried out by the parallel, scalable MD program NAMD. To verify the results obtained from computer-aided analysis, we treated Raji, Jurkat, and CT26-hCD47 cells with pyroglutamate cyclase inhibitor PQ529 during the culture process and detected SIRPα-hFc and antibody binding to PQ529 treated and untreated cells by FACS. To validate the correlation between GenSci059 binding to CD47 and the cyclization of CD47 N-terminal pyroglutamate, two human CD47 ECD fusion proteins were constructed. One had a human-derived Fc at the C-terminus (hCD47-hFc), while the other had a His tag and an enterokinase (EK) cleavage site at the N-terminal of the hCD47-hFc fusion protein (hCD47-His-EK-hFc). Binding of Gensci059 binding to indicated CD47 proteins was detected by ELISA.

### Mouse model construction

NPG (NOD-Prkdc^scid^/Il-2rg^null^) mice were subcutaneously seeded with corresponding tumor cells to establish the corresponding xerographic tumor model. When average tumor volume reached the desired volume, the animals were divided into 5 groups according to the tumor size, including the solvent control group, the GenSci059 at 5 mg/kg, 10 mg/kg and 20 mg/kg treatment groups, and the positive control (Hu5F9-G4) of 20 mg/kg treatment group, 10 mice per group. The combined effect of targeted therapy in vivo was evaluated in a Raji cells subcutaneous xenotransplantation model. The animals were divided into 5 groups including the solvent control group (vehicle), GenSci059 alone, Rituximab alone, GenSci059 + Rituximab, and Hu5F9-G4 + Rituximab, 10 mice per group. The antibodies were injected by tail vein injection, twice a week. Pharmacodynamic evaluation was carried out according to the relative tumor suppression rate (TGI). Safety evaluation was based on animal weight changes and deaths.

### Toxicity studies in NHPs

Single dose and repeat dose toxicity studies were conducted at JOINN (Suzhou) according to a written study protocol and facility standard operating procedures in compliance with Institutional Animal Care and Use Committee criteria, accepted animal welfare standards and national legal regulations on animal welfare. Single-dose toxicity study was conducted on 8 cynomolgus monkeys divided into four groups (one female and one male), where they were administered Gensci059 (50, 150 and 450 mg/kg) or blank lysate control through a single intravenous infusion on day 1. The animals were monitored continuously for 4 h and tested periodically for 22 days for various biochemical parameters. In the repeated-dose toxicity study, a total of 40 cynomolgus monkeys were randomly assigned to four groups (n = 10). The experimental groups were administered with 10 mg/kg of Gensci059 by intravenous infusion for the first administration, followed by 30 m/kg, 100 mg/kg, and 300 mg/kg of Gensci059 by intravenous infusion for the second to fifth administration, respectively. The control group was given Gensci059 blank solution at the corresponding time points. The drug was administered once a week, resulting in a total of five doses. The toxicokinetic parameters were calculated using the non-compartment analysis (NCA) on WinNonlin Professional software. Moreover, various parameters such as body weight, food intake, temperature, ECG and respiration, blood pressure, eye examination, blood cell counts, coagulation index, blood biochemical index, urinalysis, cell subsets, cytokines, immunoglobulins, complement, serum-specific antibodies, and toxicokinetic tests were assessed during the study to evaluate the safety profile of Gensci059.

### Statistical analysis

Data were shown as mean ± standard deviation (SD). Statistical significance was determined by Student’s t-test or one-way analysis of variance (ANOVA) as indicated using GraphPad Prism version 8 (CA, USA). All Student’s t-tests were two-sided under the assumption of equal variance between samples. All one-way ANOVA tests were corrected for multiple comparisons using statistical hypothesis testing. Differences were considered statistically significant if *p* < 0.05.

## Results

### Generation and validation of GenSci059 antibody

**Fig. 1:**
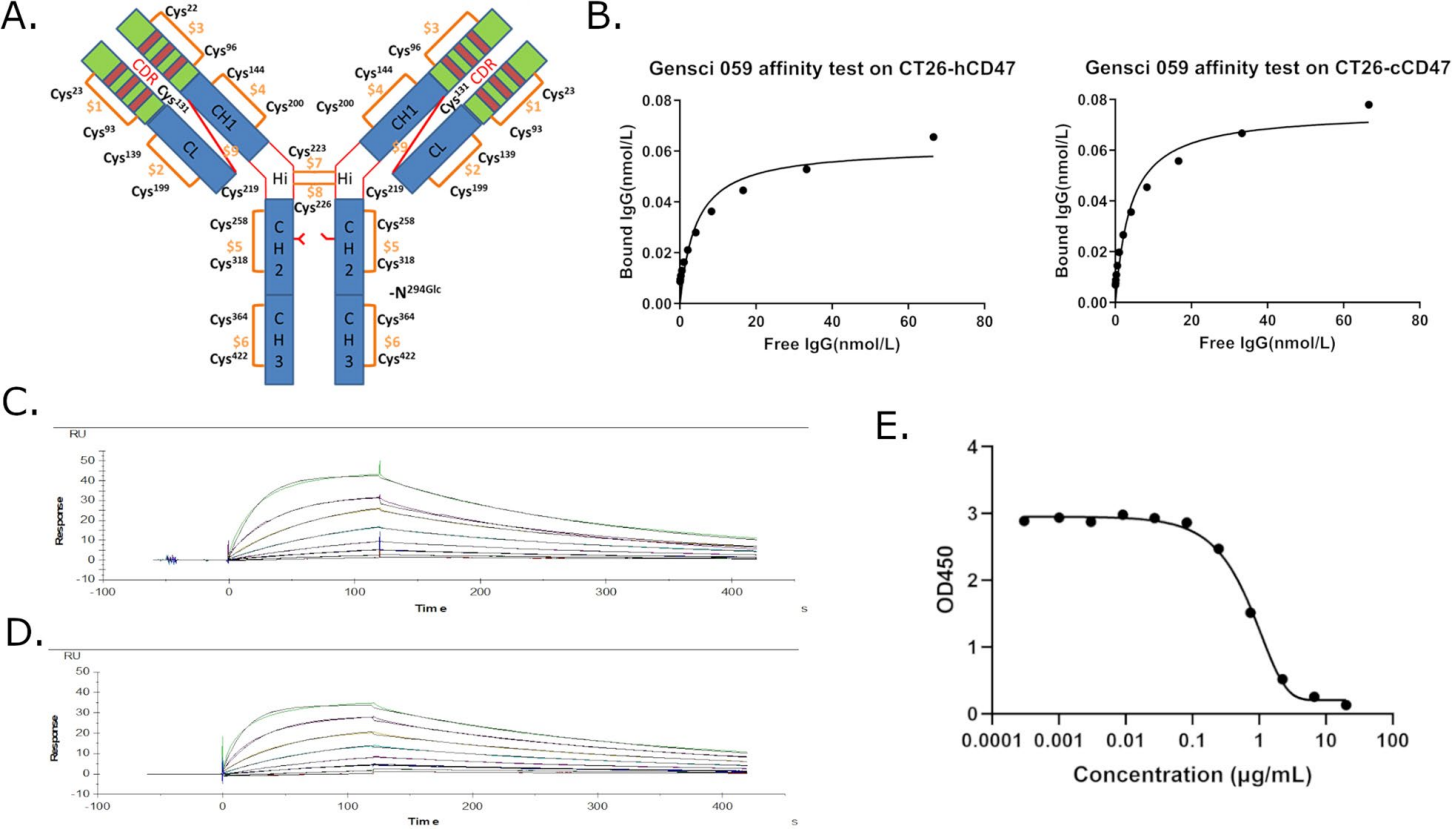
The binding characteristics of the anti-human CD47 antibody GenSci059. **A** Structure of GenSci059; **B** The cell binding affinity of GenSci059 with hCD47 and cCD47; Biacore affinity profile of Gensci059 with hCD47 **C** and cCD47 (**D**); **E** Competitive inhibition of GenSci059 inhibiting CD47 binding to SIRPα

GenSci059, a humanized monoclonal antibody against human CD47, was obtained through immunization of mice. GenSci059 is an IgG4/κ isoform, composed of two heavy chains and two light chains. Each heavy chain consists of 443 amino acids, and each light chain comprises 219 amino acids (Fig. 1A). Notably, one glycosylation site (Asn294) is present on each heavy chain. The affinity of GenSci059 with the hCD47and cCD47 on CT26 cells was measured using flow cytometry and determined to be 4.188 × 10^–9^ M and 4.005 × 10^–9^ M, respectively (Fig. 1B). The affinity of Gensci059 to CD47ECD demonstrated approximate binding ability of Gensci059 to both human and cynomolgus monkey CD47 (Fig. 1C, D, Table 1). The measured KD values were 2.05 × 10^–8^ M and 1.38 × 10^–8^ M respectively, indicating a strong binding affinity. However, Gensci059 showed no binding affinity to mouse and rat CD47 (Additional file 1: Figure S1). To explore the mechanism of action of GenSci059, the results of competitive ELISA showed that GenSci059 competitively inhibited the binding of CD47 to the SIRPα with an IC_50_ of 719 ng/mL (Fig. 1E). Collectively, these findings suggest that GenSci059 has a strong binding ability with hCD47 and has the potential to block the interaction between CD47 and SIRPα.

**Table 1:** Kinetics data of Gensci059 to extracellular domain of CD47

Species	ka (1/Ms)	kd (1/s)	KD (M)
Human CD47	2.49E + 05	5.11E-03	2.05E-08
Cynomolgus CD47	2.70E + 05	3.73E-03	1.38E-08

### GenSci059 promotes macrophage phagocytosis in vitro

**Fig. 2:**
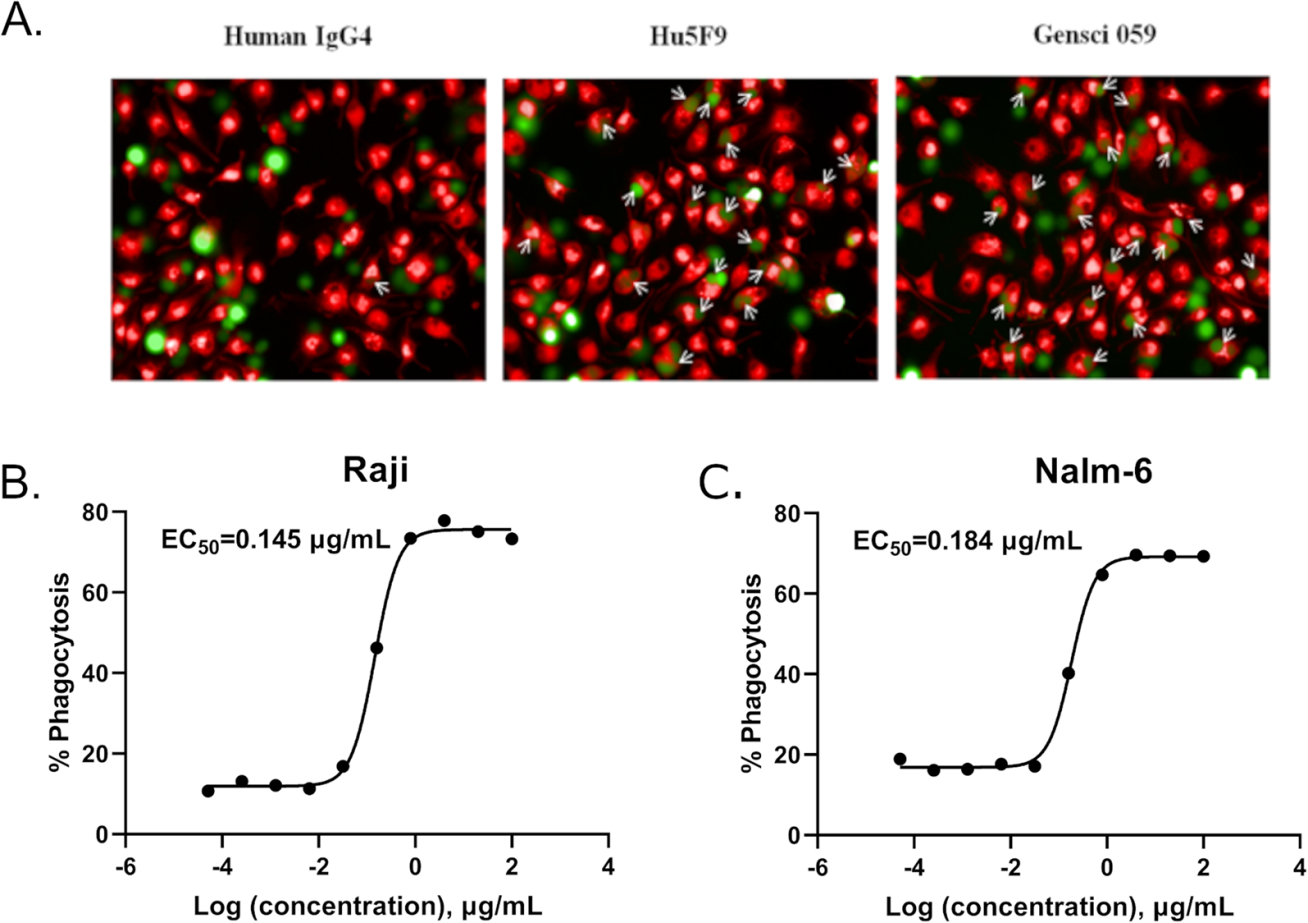
GenSci059 promoted macrophage-mediated phagocytosis of tumor cells in vitro.** A** Representative images after mouse macrophages were cocultured with Raji cell for 2 h in the presence of 20 μg/ml GenSci059, Hu5F9-G4 or control human IgG4. Tumor cells and macrophages were stained CFSE (green) and PKH-26 (red), respectively; Macrophage-mediated phagocytosis of human B-cell malignancies Raji (**B**) and Nalm-6 (**C**). Percentage phagocytosis was quantified by flow cytometry analyses

To investigate the cellular effects of GenSci059, mouse primary macrophages were utilized as effector cells, and Raji and Nalm-6 tumor cells were used as target cells. As both GenSci059 and Hu5F9-G4 belong to the human subclass of IgG4 antibodies, unrelated Human IgG4 was utilized as negative control. In this context, Hu5F9-G4 was used as a positive control. The fluorescence imaging results clearly demonstrated that Gensci059 exerted a substantial enhancement on the phagocytosis of Raji cells. Moreover, the phagocytosis indices, defined as the number of tumor cells phagocytosed per 100 macrophages, were approximately 1, 28, and 31 for Human IgG4, Hu5F9-G4, and Gensci059, respectively (Fig. 2A). Further flow cytometry assays were conducted to quantitatively the pro-phagocytic activity of GenSci059. The dose–response curve indicated stimulatory effects of GenSci059 in promoting the phagocytosis of Raji and Nalm-6 in mouse macrophages, with an EC_50_ of 0.145 μg/mL and 0.184 μg/mL, respectively (Fig. 2B, C). In vitro migration assay revealed that GenSci059 significantly decreased HL-60 and Kasumi-1 migration (Additional file 1: Figure S2).

### Validation of GenSci059 in toxicity risk

**Fig. 3:**
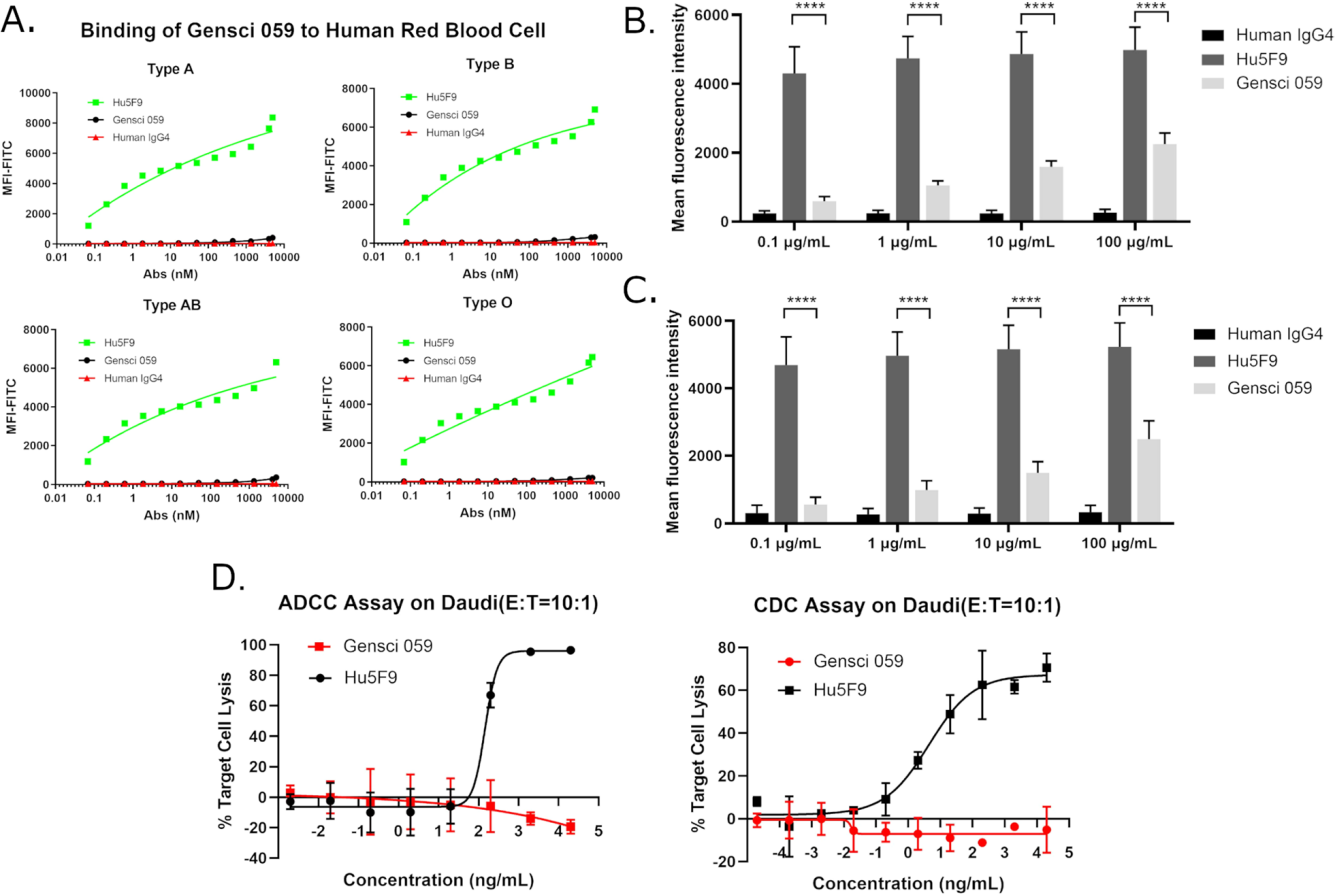
Safety evaluation results of GenSci059. **A** GenSci059 and the binding test results of different blood groups of red blood cells; **B** Four concentrations of GenSci059 were combined with human PBMCs; **C** GenSci059 binds to human platelets; **D** ADCC and CDC effects of GenSci059

To investigate the safety of GenSci059, binding experiments were conducted with human red blood cells (hRBCs). The control antibody, Hu5F9-G4, exhibited a high binding rate with hRBCs across all four blood types, which increased with the concentrations regardless of blood type. In contrast, GenSci059 showed minimal binding to hRBCs and only weakly bound at high concentrations (Fig. 3A). To better predict the safety risk in clinical trials, binding activity was also conducted with peripheral blood mononuclear cells (PBMC) and platelets (PLT). Results indicated that GenSci059 had weaker binding to PBMC and PLT than Hu5F9-G4 at all four detected concentrations (Fig. 3B, C), indicative of a lower safety risk than Hu5F9-G4. To further evaluate the antibody-mediated cytotoxicity of GenSci059, ADCC and CDC experiments were performed using Daudi as the target cell and Rituxan as the positive control antibody. The results showed that Rituxan had a normal dose–response curve, whereas GenSci059 had no ADCC or CDC at the concentrations observed (Fig. 3D). These findings suggest that the safety of GenSci059 is better than that of the control antibody Hu5F9-G4.

### Binding of GenSci059 to hCD47 under different conditions

**Fig. 4:**
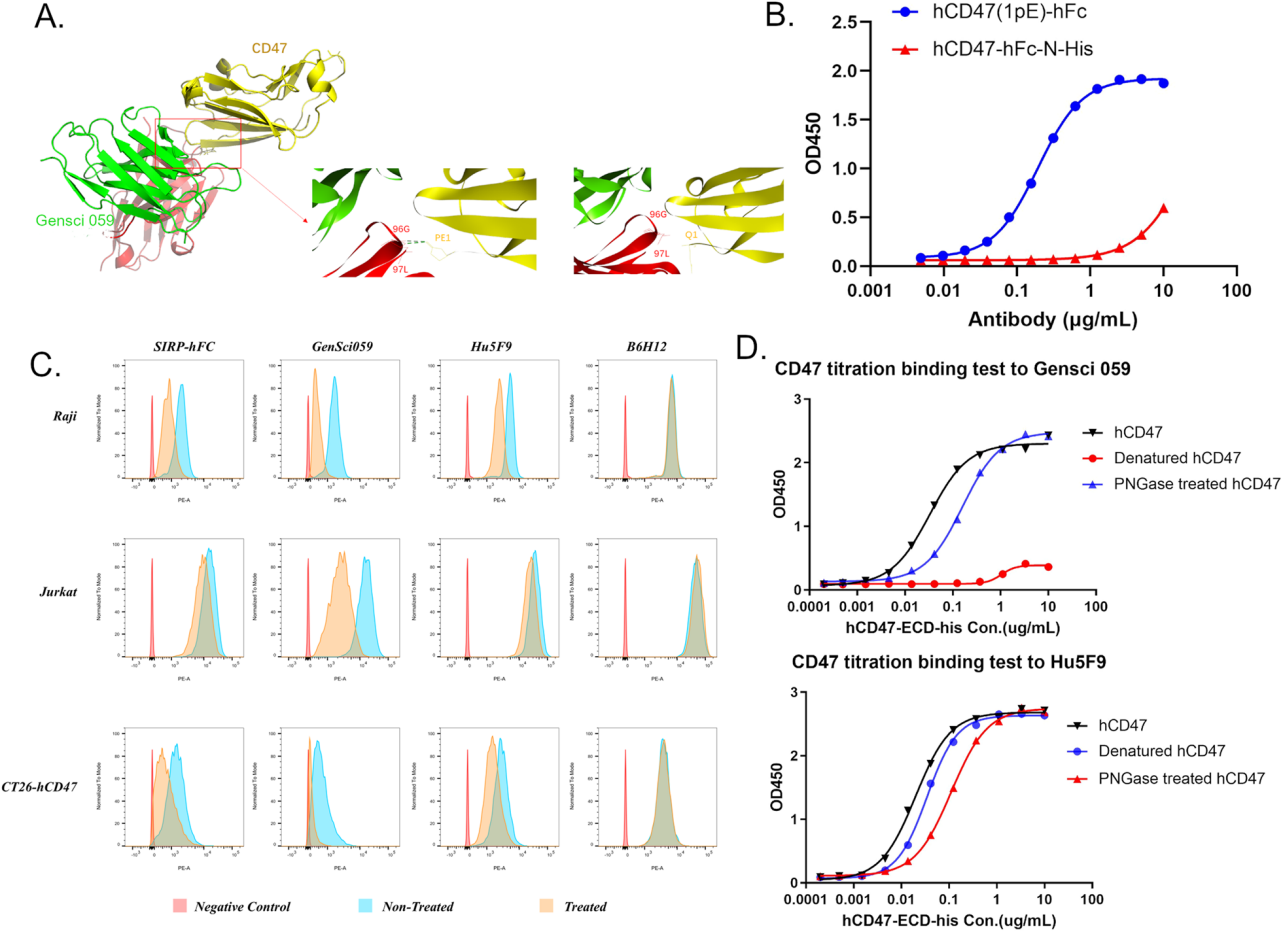
Unique binding pattern of GenSci059 to CD47. **A** Protein docking analysis of GenSci059 binding to CD47; **B** Effect of N-terminal pyroglutamate cyclization on the binding of GenSci059 to CD47; **C** Pyroglutamate cyclase inhibitor impaired the binding of GenSci059 to the cells; **D** Effect of CD47 spatial conformation and glycosylation modification of the binding epitope on the binding of GenSci059 to CD47

The mechanism underlying the low binding activity of GenSci059 to erythrocytes was investigated using antibody protein simulation docking to identify the binding mode of GenSci059 to CD47. The docking results revealed that CD47 with N-terminal pyroglutamate (CD47-PE1) exhibited higher binding affinity to GenSci059 compared to CD47 with N-terminal glutamate (CD47-Q1), with an energy difference of 47.2 kcal/mol (Fig. 4A). This result suggests that CD47 N-terminal pyroglutamate, located at the binding epitope for GenSci059, plays a crucial role. The ELISA results revealed the N-terminal His tag of hCD47-His-EKase-hFc inhibits the cyclization of the N-terminal pyroglutamate. The obtained hCD47-hFc-N-His fusion protein was subjected to deglycosylation using Peptide-N-Glycosidase F (PNGase F). Subsequently, the His-tag was removed using EK, and the resulting products were characterized together with hCD47-hFc by LC–MS based peptide mapping. The results showed that hCD47-hFc and hCD47-hFc-N-His displayed 100% and 25.85% pyroglutamate modification of N-terminal peptide, respectively (peptide with N-terminal pyroglutamate: 746.4 Da; peptide without N-terminal pyroglutamate: 763.4 Da) (Additional file 1: Figure S3). ELISA also showed strong binding of GenSci059 to hCD47-hFc and weak binding to uncyclized hCD47-hFc (Fig. 4B). Moreover, when Jurkat cells, Raji cells, and CT26 cells overexpressing CD47 were treated the pyroglutamate cyclase inhibitor PQ529, FACS analysis showed that PQ529 impaired the binding of SIRPα-hFc, GenSci059, and Hu5F9-G4 to the cells. The results indicated that GenSci059 binding is influenced specifically by the cyclization of the N-terminal pyroglutamate of CD47 (Fig. 4C). To further investigate the relationship between GenSci059 and CD47 in terms of CD47 spatial conformation and glycosylation modification of the binding epitope, hCD47 protein was subjected to thermal denaturation and glycosidase hydrolysis. We observed that GenSci059 bound normally to non-denatured CD47 but not to denatured CD47 (Fig. 4D). Additionally, glycosidase treatment only slightly inhibited the binding of GenSci059 and Hu5F9-G4 to CD47. In contrast, the control antibody Hu5F9-G4 exhibited normal binding to both denatured and non-denatured CD47. Based on these findings, it is evident that the binding of GenSci059 to CD47 depends on the cyclization of N-terminal pyroglutamate and spatial conformation of CD47, which account for the enhanced antibody specificity and improved safety for GenSci059.

### GenSci059 exhibited effective antitumor activity

**Fig. 5:**
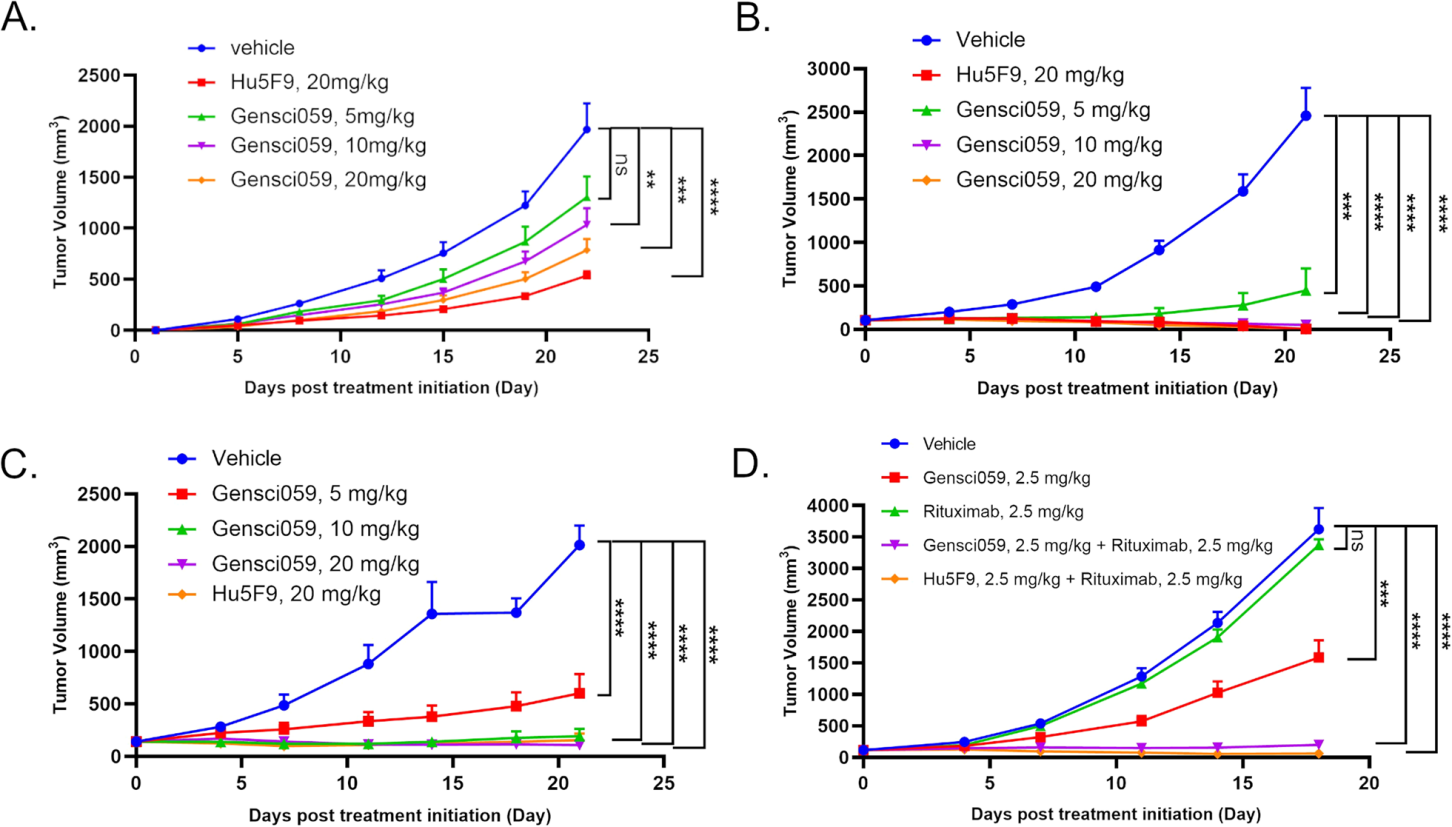
GenSci059 anti-tumor activity results in tumor-bearing mouse models. PDX model results of subcutaneous xenotransplantation of Human non-Hodgkin lymphoma cell Raji (**A**), HL-60 (**B**) and small cell lung cancer cell LU2514 (**C**); **D** Inhibitory effect of GenSci059 combined with Rituximab on Raji tumor growth. *P* values for tumor volume analysis apply to the final time point as indicated in graphs and were calculated by Student’s t test, *p < 0.05, **p < 0.01, ***p < 0.001, ****p < 0.0001

In a PDX model of subcutaneous xenotransplanted human non-Hodgkin lymphoma Raji cell, GenSci059 effectively inhibited tumor growth in a dose-dependent manner with TGI ranging from 49.49% to 60.90% (Fig. 5A, Additional file 1: Figure S4). At the same dose, GenSci059 show slightly more potent inhibition in tumor growth than the positive control Hu5F9-G4 (87.0% vs 79.6%). In the human acute myeloid leukemia HL-60 cell and small cell lung cancer LU2514 cell subcutaneous xenograft tumor model, GenSci059 also showed significant tumor inhibition ability with TGI ranging from 79.1% to 100% and 51.2% to 87.0%, respectively (Fig. 5B, C). Importantly, GenSci059 was well tolerated and exhibited no significant toxicity during administration. The combination of GenSci059 with rituximab in a subcutaneous xenotransplantation model for human lymphoma was also evaluated. Results showed that the combination of GenSci059 with rituximab significantly inhibited Raji model tumor growth, with a TGI of 94.6% compared to 55.1% for GenSci059 monotherapy and 8.0% for rituximab monotherapy (Fig. 5D), indicating that GenSci059 can be combined with antibodies against other tumor antigens to broaden the application of anti-CD47 cancer immunotherapy. Overall, these findings suggest that GenSci059 has strong tumor inhibition ability and has the potential as an anti-tumor candidate.

### GenSci059 has a favorable safety profile in non‑human primates

To evaluate the potential risk of GenSci059 in humans, toxicity studies were conducted in cynomolgus monkeys. In the single-dose toxicity study, GenSci059 was administered as single intravenous infusions of 50, 150, and 450 mg/kg to cynomolgus monkeys, and they were observed for 22 days after administration. During the observation period, the erythrocyte count decreased by a maximum of 26.0%, and hemoglobin decreased by a maximum of 26.3% (high dose group). The results mainly showed a decrease in erythroid-related parameters and an increase in reticulocytes, fibrinogen, and total bilirubin, which returned to pre-dose levels on day 22. The maximum tolerated dose (MTD) of GenSci059 was found to be 450 mg/kg under these conditions. In the one-month repeated-dose toxicity study (Table 2), no abnormal changes that were clearly associated with GenSci059 or of toxicological significance were observed. Hematologic examinations showed a decrease in erythrocyte-related parameters, with a maximum decrease in mean red blood cell count of 19%, a maximum decrease in mean hemoglobin of 17%, and elevated reticulocytes in all dosing groups, but without toxicological significance. Histopathological examination showed no significant donor-related pathological changes in animals. Under the test conditions, the NOAEL was 10 + 300 mg/kg, as no significant toxic reaction dose was observed.

**Table 2:** Toxicokinetic parameters of Gensci059 in cynomolgus monkeys following multiple-dose administration

	1st dose			2nd dose			4th dose			
	C_max_ (mg/mL)	AUC_last_ (h·mg/mL)	AUC_inf_ (h·mg/mL)	C_max_ (mg/mL)	AUC_last_ (h·mg/mL)	AUC_inf_ (h·mg/mL)	C_max_ (mg/mL)	AUC_last_ (h·mg/mL)	AUC_inf_ (h·mg/mL)	AF (AUC_4th/2nd_)
Low dose	0.19 ± 0.01	3.1 ± 0.48	3.1 ± 0.48	0.73 ± 0.08	27.33 ± 2.88	28.53 ± 2.94	0.77 ± 0.31	34.12 ± 21.36	36.34 ± 22.96	1.25
Middle dose	0.19 ± 0.04	3.11 ± 0.54	3.11 ± 0.54	2.46 ± 0.23	149.24 ± 22.96	173.14 ± 28.15	2.92 ± 0.32	155 ± 61.03	234.92 ± 132.32	1.04
High dose	0.21 ± 0.02	3.51 ± 0.58	3.51 ± 0.58	8.89 ± 0.71	521.26 ± 60.07	737.03 ± 174.88	7.98 ± 1.31	556.2 ± 178.94	1001.69 ± 503.81	1.07

*C*_*max*_ maximum observed concentration; *AUC* Area under the concentration–time curve; *AUC*_*last*_ AUC to the Last Measurable Concentration; *AUC*_*inf*_ AUC from time 0 to infinity; *AF* Accumulation factor

## Discussion

CD47 is a transmembrane glycoprotein that is highly expressed by tumor cells, enabling them to evade immune surveillance mediated by macrophages through the CD47-SIRPα complex. Monoclonal antibodies or fusion proteins targeting CD47 have the potential to disrupt this “don’t eat me” signal, facilitating the macrophages mediated phagocytosis of tumor cells and promoting an immune response against the tumor [12]. Several molecules targeting this axis are currently being evaluated in clinical trials [12, 27, 29, 30]. Our results demonstrated that GenSci059, a humanized anti-human CD47 monoclonal antibody, selectively binds to the pyroglutamate cycled CD47 and consequently blocks the CD47/SIRPα axis signaling pathway by inhibiting the interaction between CD47 and SIRPα. We showed that GenSci059 could induce the phagocytosis of AML cells by macrophages in vitro, and exhibits effective antitumor activity in a tumor bearing mice model. Additionally, the safety evaluation of GenSci059 indicated no ADCC or CDC activities as well as low blood toxicity. These results collectively suggest that GenSci059 represents a promising monoclonal antibody targeting CD47 with an ideal safety profile.

CD47 plays a crucial role in hematological malignancies, including acute myeloid leukemia (AML), B- and T-cell acute leukemia, and non-Hodgkin's lymphoma. The use of anti-CD47 antibodies has shown promising potential in promoting tumor cell elimination through various mechanisms, including the enhancement of phagocytosis by macrophages and the augmentation of non-phagocytic killing by neutrophils and natural killer (NK) cells. Our in vitro studies demonstrate that GenSci059 effectively blocks the interaction between CD47 and its inhibitory receptor SIRPα, leading to a dose-dependent promotion of tumor cell phagocytosis by both mouse and human macrophages. Interestingly, GenSci059 induces neither antibody-dependent cell-mediated cytotoxicity (ADCC) nor complement-dependent cytotoxicity (CDC). In tumor-bearing mouse models, treatment with GenSci059 at doses ranging from 5 to 20 mg/kg exhibited potent inhibition of subcutaneous tumors, resulting in tumor inhibition rates ranged from 30.3% to complete tumor regression. These pharmacodynamic effects were comparable to those observed in the positive control Hu5F9-G4. Notably, in the Raji model, the combined administration of GenSci059 with Rituximab demonstrated superior tumor growth inhibition compared to either agent alone. Furthermore, GenSci059 exhibited a favorable safety profile in the tested animal models, as it was well tolerated at the doses studied. These findings suggest that GenSci059 holds promise as a potential therapeutic agent with potent anti-CD47 activity and a favorable safety profile.

CD47 is not only present on tumor cells but also expressed on the surface of erythrocytes, platelets, and hematopoietic stem cells. The CD47-SIRPα axis plays a crucial role in regulating the clearance and homeostasis of these blood cells under normal physiological conditions [36–38]. Therefore, one of the major challenges in the development of CD47 antibody-based anticancer drugs is to maximize the killing of tumor cells while ensuring the protection of red blood cells. Achieving this balance is a central and pressing issue in CD47 antibody development. Previous studies have shown a high incidence of grade 3 and 4 treatment-related adverse events, including anemia, neutropenia, and thrombocytopenia, in patients with myelodysplastic syndromes (MDS) and acute myeloid leukemia (AML) with the CD47 antibody Hu5F9-G4 even at low doses [27]. In our study, we conducted a large-scale screening and identified GenSci059 that exhibits weak binding to erythrocytes. We further verified the binding of GenSci059 to hRBC, PBMC and PLT in various blood samples. Although GenSci059 exhibited binding to these cells, its binding capacity was found to be weaker compared to the positive control Hu5F9-G4. In addition, our in vivo and in vitro studies demonstrated that GenSci059 did not pose a significant risk of cytokine release. This indicates that GenSci059 has a lower likelihood of inducing anemia, leukopenia, and thrombocytopenia compared to the positive control drug, suggesting a better safety profile for GenSci059. These findings highlight the potential of GenSci059 to minimize the risk of blood-related adverse events while maintaining its therapeutic efficacy as a CD47-targeted therapy.

A number of CD47 antibodies have been designed with weak binding to CD47 on RBCs. However, the mechanism by which the antibodies are associated with RBC hemagglutination is not known. In the present study, we observed that the binding of Gensci059 to human RBCs was negligible. A potential explanation for the unique functional properties of Gensci059 may due to its unique binding mode to CD47. AK117, a novel CD47-targeting antibody, does not induce hemagglutination of RBCs while effectively blocking CD47 on tumor cells [39]. Spatial structure analysis based on molecular simulations suggests that the AK117/CD47 complex adopts a unique binding conformation, allowing AK117 to bind to only one CD47 on RBCs, which may contribute to the hemagglutination resistance of AK117. In contrast, the Y-shaped conformation of the Hu5F9-G4/CD47 complex may contribute to the binding of Hu5F9-G4 to two independent CD47 on RBCs, potentially leading to hemagglutination. Although the specific binding conformation of Gensci059 to CD47 was not explored in this study, the altered spatial conformation of CD47 could almost completely block the binding of Gensci059 to CD47, suggesting that the binding of Gensci059 to CD47 is spatial conformation dependent. The binding conformation of Gensci059/CD47 and Hu5F9-G4/CD47 complexes is different, which may be similar to the way AK117 and SRF231 binds to RBCs [39, 40]. Excessive activation of pyroglutamate cyclase is a key factor leading to tumor evasion of macrophages via the CD47-SIRPα signaling pathway. This over-activation may lead to the cyclization of CD47 N-terminal pyroglutamate on the surface of tumor cells, and thus enhances the binding capacity to SIRPα [31, 41]. The distribution of pyroglutamate cyclase varies between cells and tissues [41], and high levels of pyroglutamate cyclase are associated with decreased overall survival in most cancer types (http://gepia.cancer-pku.cn/detail.php?gene=QPCT), suggesting that membrane proteins of cancer cells may have higher levels of pyroglutamate cyclization relative to normal tissues. Whereas the binding of Gensci059 to CD47 depends on the level of CD47 N-terminal pyroglutamate cyclization, the high level of pyroglutamate cyclization in cancer cells may enhance their binding to Gensci059. The binding mechanism of TJC4 to CD47 has also been investigated, and the structure of the TJC4-CD47 complex is a new conformational epitope with head-to-head linear binding. Erythrocytes are characterized by a high degree of glycosylation of membrane proteins, and N-linked glycosylation sites near two key epitopes on the CD47 protein prevent the binding of TJC4 to human RBCs [42]. The effect of protein glycosylation on the binding of Gensci059 to CD47 was similarly explored in this study, and it was found that the binding of Gensci059 to hCD47 was less affected by protein glycosylation.

Given that GenSci059 is an antibody-based drug candidate, it was essential to determine the appropriate animal species for non-clinical pharmacotoxicological testing studies. To assess the binding affinity of GenSci059 with CD47 among different species, experiments were conducted. We found that GenSci059 binds to CD47 in human and cynomolgus monkeys but not in mice. Based on these findings, key toxicological studies were conducted in the relevant animal species cynomolgus monkeys. We revealed that the exposure to GenSci059 was a maximum tolerated dose (MTD) of 450 mg/kg. This indicates a favorable safety profile for GenSci059. Notably, the MTD was found to be 1800 times the effective dose observed in the pharmacodynamic assay of GenSci059 (0.25 mg/kg). Furthermore, the administration of GenSci059 led to a decrease in erythroid-related parameters and an increase in reticulocytes, fibrinogen, and total bilirubin, which returned to pre-dose levels by day 22. No safety concerns such as bleeding due to reductions in platelets were observed in the animals. In the one-month repeated-dose toxicity study, no significant toxic effects were observed. Under the test conditions, the no observed adverse effect level (NOAEL) was determined to be 10 + 300 mg/kg, which was 10 times higher than the anticipated clinical dose of 30 mg/kg for the same target drug and 120 times the effective dose observed in the GenSci059 mouse pharmacodynamic assay (0.25 mg/kg). Moreover, considering exposure, the NOAEL dose in the one-month toxicology test in cynomolgus monkeys was 4.6 times higher than the exposure observed in the clinical test of the same target product, Hu5F9-G4, further supporting the favorable safety profile of GenSci059. These comprehensive findings from the non-clinical studies provide crucial insights for the future clinical development of GenSci059 as a promising drug candidate. Further investigations and clinical trials are warranted to fully evaluate the safety and effectiveness of GenSci059 in patients with hematological malignancies.

Our study acknowledges areas that merit further investigation and strengthening: (1) The precise anti-tumor mechanism of GenSci059 remains to be fully elucidated. Additionally, the impact of GenSci059 on immune cell function, specifically on human T, B, and NK lymphocytes, remains an aspect to be explored further. (2) This study primarily demonstrated the capability of GenSci059 to inhibit tumor growth. However, the potential inhibitory effect of GenSci059 on metastasis and relapse of AML or other cancers has yet to be explored comprehensively. (3) Our study primarily concentrated on assessing the impact of GenSci059 on hematologic adverse events. The potential effects of GenSci059 on cellular functions of other cell types remain unclear. Thus, a more extensive exploration of the safety profile of GenSci059 is warranted.

## Conclusion

In conclusion, our study highlights the potential of GenSci059 as a superior anti-CD47 monoclonal antibody. Its selective binding to pyroglutamate cycled CD47 effectively blocks the CD47/SIRPα axis signaling pathway, leading to enhanced phagocytosis of tumor cells by macrophages. Furthermore, GenSci059 exhibits potent antitumor activity and demonstrates a favorable safety profile.

### Supplementary Information

**Additional file 1: Figure S1.** Biacore affinity profile of Gensci059 with Mouse CD47 (A) and Rat CD47 (B). **Figure S2.** GenSci059 significantly decreased HL-60 (A) and Kasumi-1 (B) migration. **Figure S3.** LC-MS based peptide mapping results of hCD47-hFc-N-His (A) and hCD47-hFc (B). **Figure S4.** Photograph of the tumors extracted from the PDX model of subcutaneous xenotransplantedRaji cell for evaluating the combination effect of GenSci059 with rituximab.

## Data Availability

The data supporting the findings of this study are available from GeneScience Pharmaceuticals under license and are not publicly available due to restrictions. However, the authors are able to provide the data upon reasonable request.
