# Supplementary material for: Gentulizumab, a novel anti-CD47 antibody with potent antitumor activity and demonstrates a favorable safety profile
Source: J Transl Med. 2024 Mar 1;22:220. doi: 10.1186/s12967-023-04710-6 (PMC10905820; doi:10.1186/s12967-023-04710-6)

Figure S1. Biacore affinity profile of Gensci059 with Mouse CD47 (A) and Rat CD47 (B).

A.

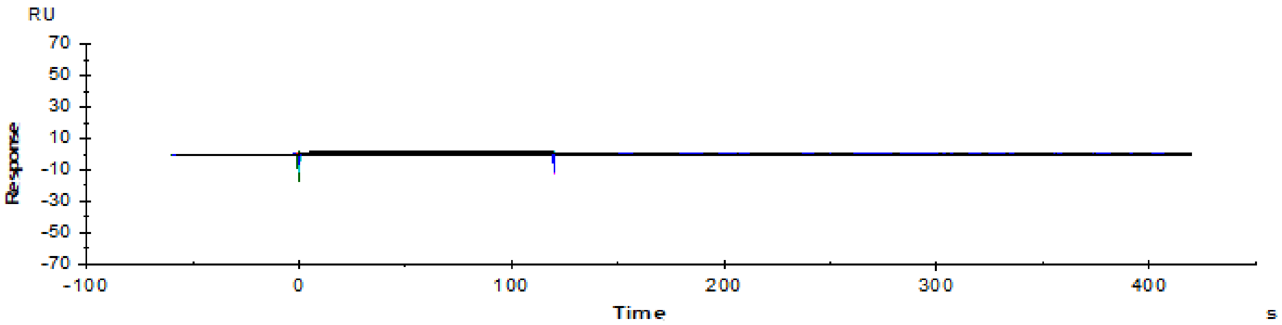

B.

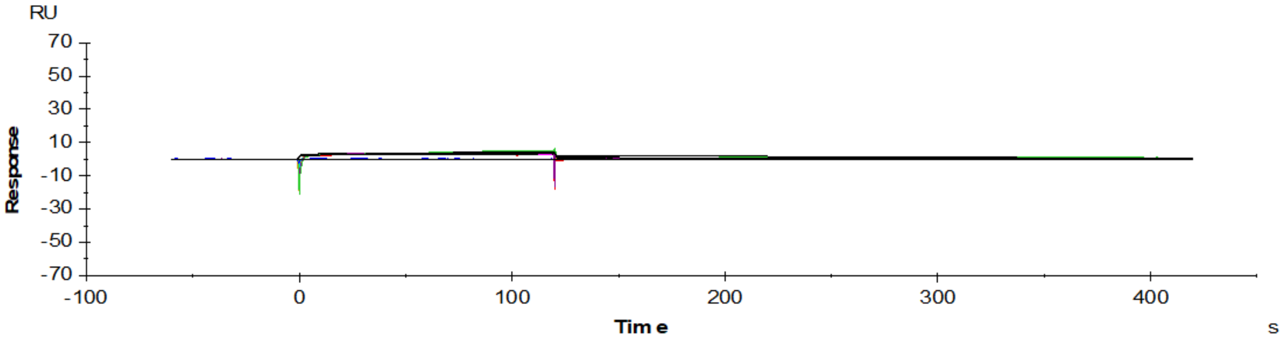

Figure S2. GenSci059 significantly decreased HL-60 (A) and Kasumi-1 (B) migration

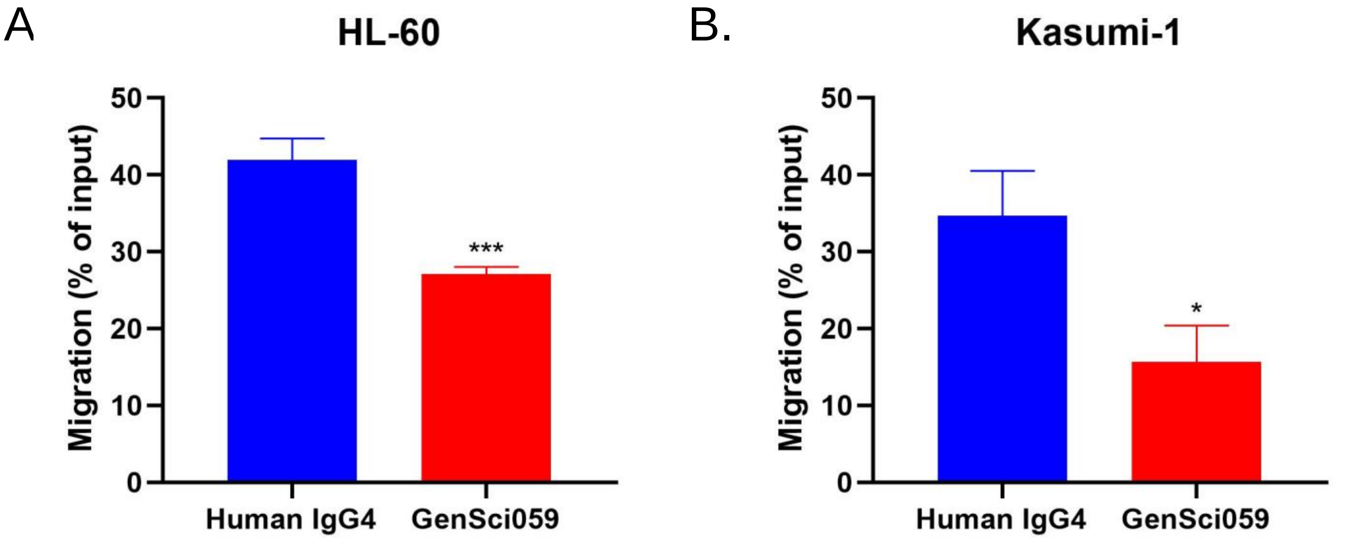

Figure S3. LC-MS based peptide mapping results of hCD47-hFc-N-His (A) and hCD47-hFc (B).

A.

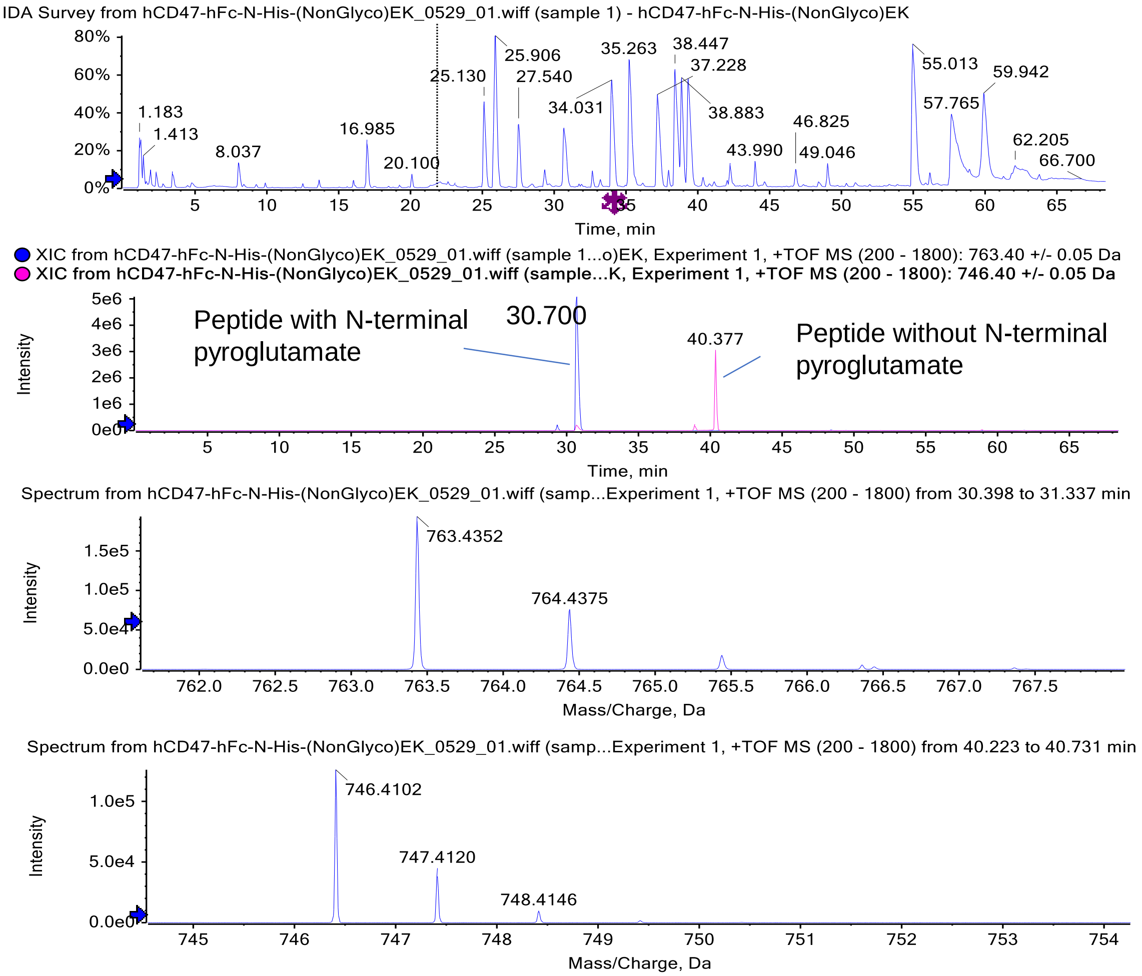

B.

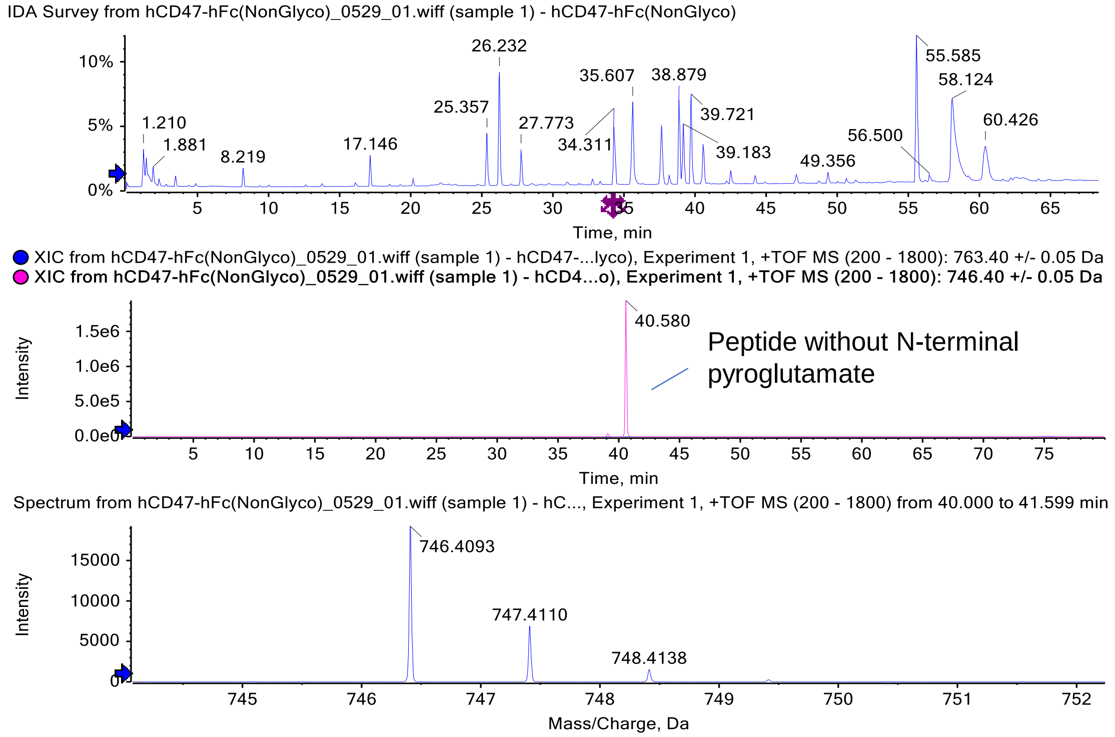

Figure S4. Photograph of the tumors extracted from the PDX model of subcutaneous xenotransplanted Raji cell for evaluating the combination effect of GenSci059 with rituximab.

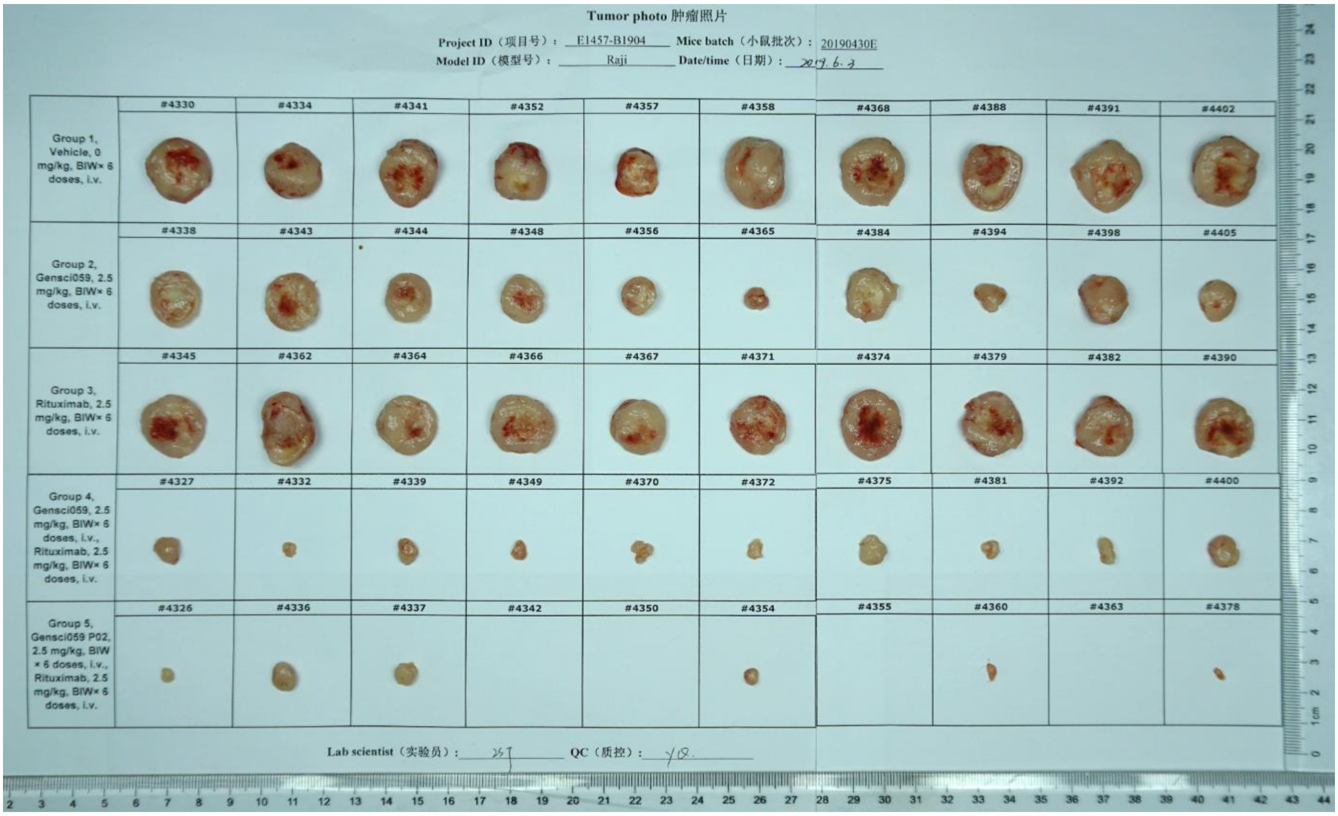

Supplement: Supplementary file 1 — Additional file 1: Figure S1. Biacore affinity profile of Gensci059 with Mouse CD47 (A) and Rat CD47 (B). Figure S2. GenSci059 significantly decreased HL-60 (A) and Kasumi-1 (B) migration. Figure S3. LC-MS based peptide mapping results of hCD47-hFc-N-His (A) and hCD47-hFc (B). Figure S4. Photograph of the tumors extracted from the PDX model of subcutaneous xenotransplantedRaji cell for evaluating the combination effect of GenSci059 with rituximab. [file 12967_2023_4710_MOESM1_ESM.pdf]
